# The Frequency of Different Risk Factors for Lower Back Pain in a Tertiary Care Hospital

**DOI:** 10.7759/cureus.3183

**Published:** 2018-08-22

**Authors:** Faleha Zafar, Yusaf F Qasim, Muhammad Umer Farooq, Ibrahim Shamael, Inayat U Khan, Danish Hassan Khan

**Affiliations:** 1 Neurology, Shifa International Hospital, Islamabad, PAK; 2 Medicine, Shifa International Hospital, Islamabad, PAK; 3 Shifa College of Medicine, Shifa Tameer-e-Millat University, Islamabad, PAK; 4 Neurology, Shifa International Hospital, Islamabad , PAK; 5 Research Department, Shifa International Hospital, Islamabad, PAK

**Keywords:** lower back pain, risk factors, cross-sectional study, islamabad, pakistan

## Abstract

Background and purpose

Lower back pain is an extremely common health problem and causes more global disability than any other condition. Moreover, it causes an enormous economic burden in both developed and developing countries. The aim of this study is to determine the frequency of different risk factors for lower back pain in a tertiary care centre in Islamabad.

Methods

A cross-sectional study was conducted at Shifa International Hospital’s neurosurgery and neurology outpatient department from September 2016 to February 2017. A total of 375 patients with lower back pain were interviewed regarding risk factors.

Results

Among the 375 patients, the majority were men (51.7%, n = 194). The mean patient age was 42.05 ± 15.35 years (mean ± standard deviation); most of the patients belonged to the 21- to 40-year-old age group (48%, n = 180). The majority (78.4%) had chronic back pain. Lower back pain was found to be predominant in housewives (30.1%, n = 113), followed by those with office jobs (18.1%, n = 68), private jobs (i.e., truck drivers, shopkeepers) (14.7%, n = 55), and healthcare workers (12.3%, n = 46). In terms of work schedule, 51.2% of patients reported working around 41–50 hours per week. The major risk factors identified were lack of exercise (76.3%, n = 286), use of soft foam mattress (52.0%, n = 195), prolonged sitting (50.4%, n = 189), lifting heavy weight (48.5%, n = 182), bending or twisting (41.6%, n = 156), sleep disorder (41.6%, n = 156), anxiety (39.5%, n = 148), hypertension (32.3%, n = 121), and depression (28.8%, n = 108).

Conclusions

Our study concludes that lower back pain is a multifactorial phenomenon. Age, gender, profession, working hours, comorbid conditions, trauma, lifestyle, and stresses in life all play a role in its causation. Increasing physical activity and modifying lifestyle are suggested to prevent this major health issue.

## Introduction

Lower back pain (LBP) is an extremely common health problem [1-4]. Lower back pain causes more global disability than any other condition. Disability-adjusted life years (DALYs) increased from 58.2 million in 1990 to 83 million in 2010 [5]. It is causing an enormous economic burden in both developed and developing countries [6-8].

Lower back pain is defined as a nonspecific condition that refers to complaints of acute or chronic pain and discomfort in the area between the lower posterior margin of the ribcage and the horizontal gluteal fold [9].

There are numerous diverse causes of lower back pain including occupational, behavioural, socio-economic, and metabolic. Although there are some studies regarding this subject, there is still a need for more studies in developing countries like Pakistan, so that the frequency of different risk factors associated with lower back pain can be determined. This will eventually help the clinicians manage the patient as a whole instead of merely prescribing analgesics or advising exercise.

## Materials and methods

A cross-sectional study was conducted at the Shifa International Hospital's neurosurgery and neurology outpatient department (OPD) from September 2016 to February 2017. All patients above the age of 18 years presenting to the neurosurgery and neurology OPD with a complaint of lower back pain were included in the study. An informed consent was obtained from all participants. Those who refused to give consent were excluded from our study. The patients were selected using non-randomized convenient sampling. A sample size of 375 was estimated, using an anticipated population precision of 58% [10], with an absolute precision of 0.5, and a confidence level of 95%.

The selected patients were interviewed regarding their occupation, working hours, hostile job environment, job insecurity, sleep disorders, depression, anxiety, and comorbidities like obesity, hypertension, coronary artery disease, smoking, and alcohol consumption. A data collection form, in English, was prepared and used to document the patient’s age, sex, duration of symptoms, and risk factors for lower back pain mentioned above. The results were analyzed using Statistical Package for Social Sciences (SPSS, IBM, Armonk, NY) software version 21. A descriptive analysis was performed on the variables.

## Results

**Figure 1:**
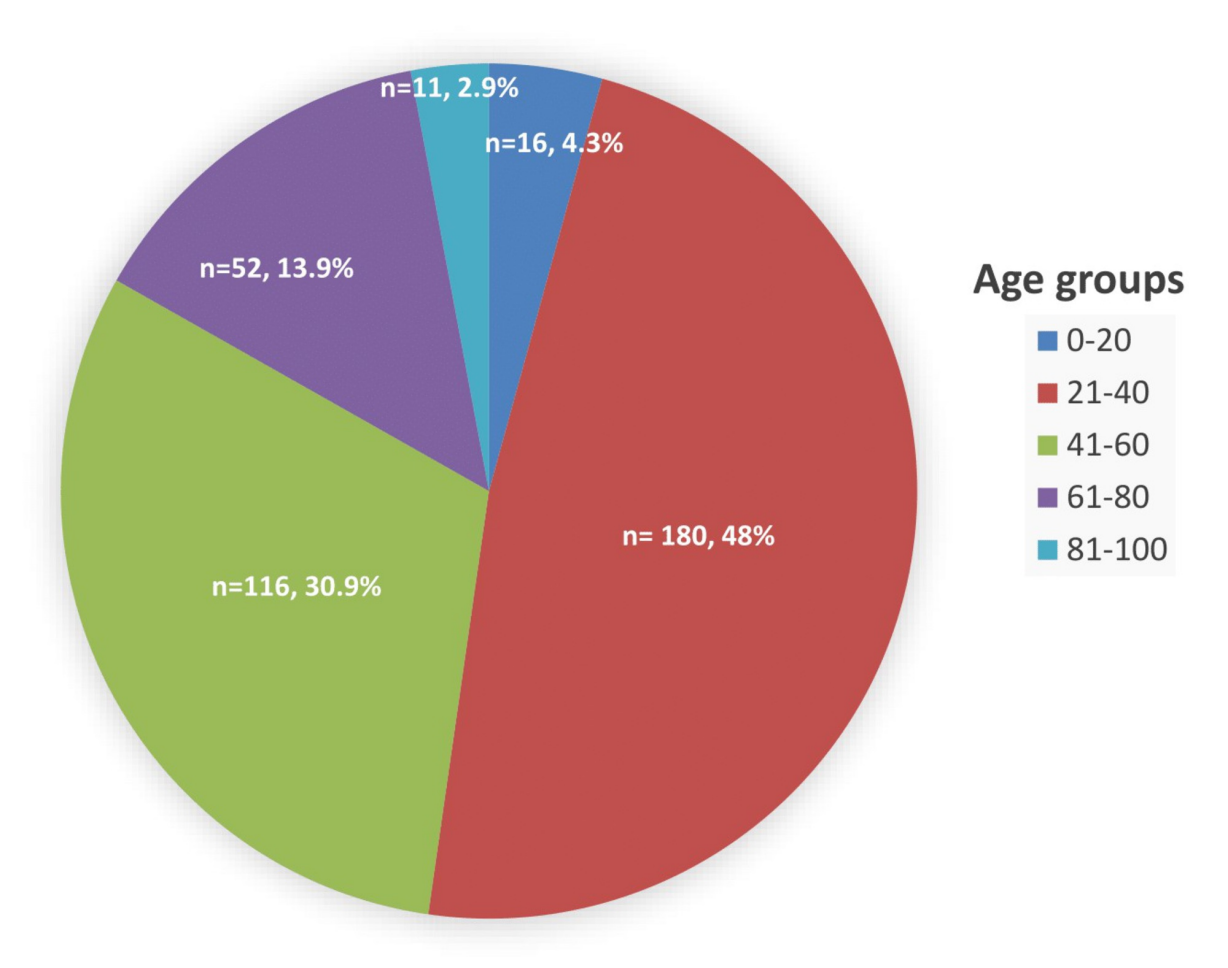
Distribution of lower back pain among various age groups.

Among the 375 patients, majority were male (51.7%, n = 194) and 181 (48.3%) were female. The mean patient age was 42.05 ± 15.35 years (mean ± standard deviation). While most of the patients, 296 of 375, were 21–60 years old (78.9%), the majority of the patients belonged to the 21–40 years old age group (48%, n = 180) as shown in Figure 1.

**Figure 2:**
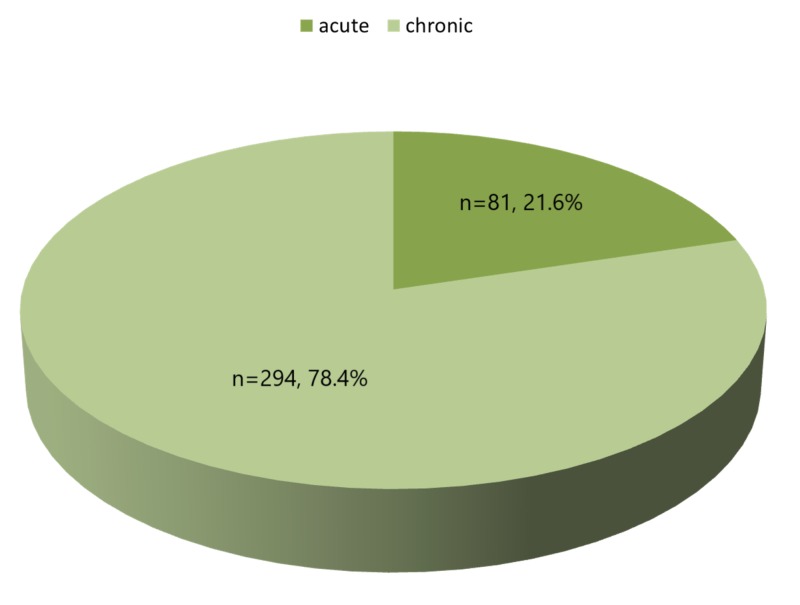
Distribution between acute and chronic back pain.

The majority (78.4%) had chronic back pain as shown in Figure 2.

**Figure 3:**
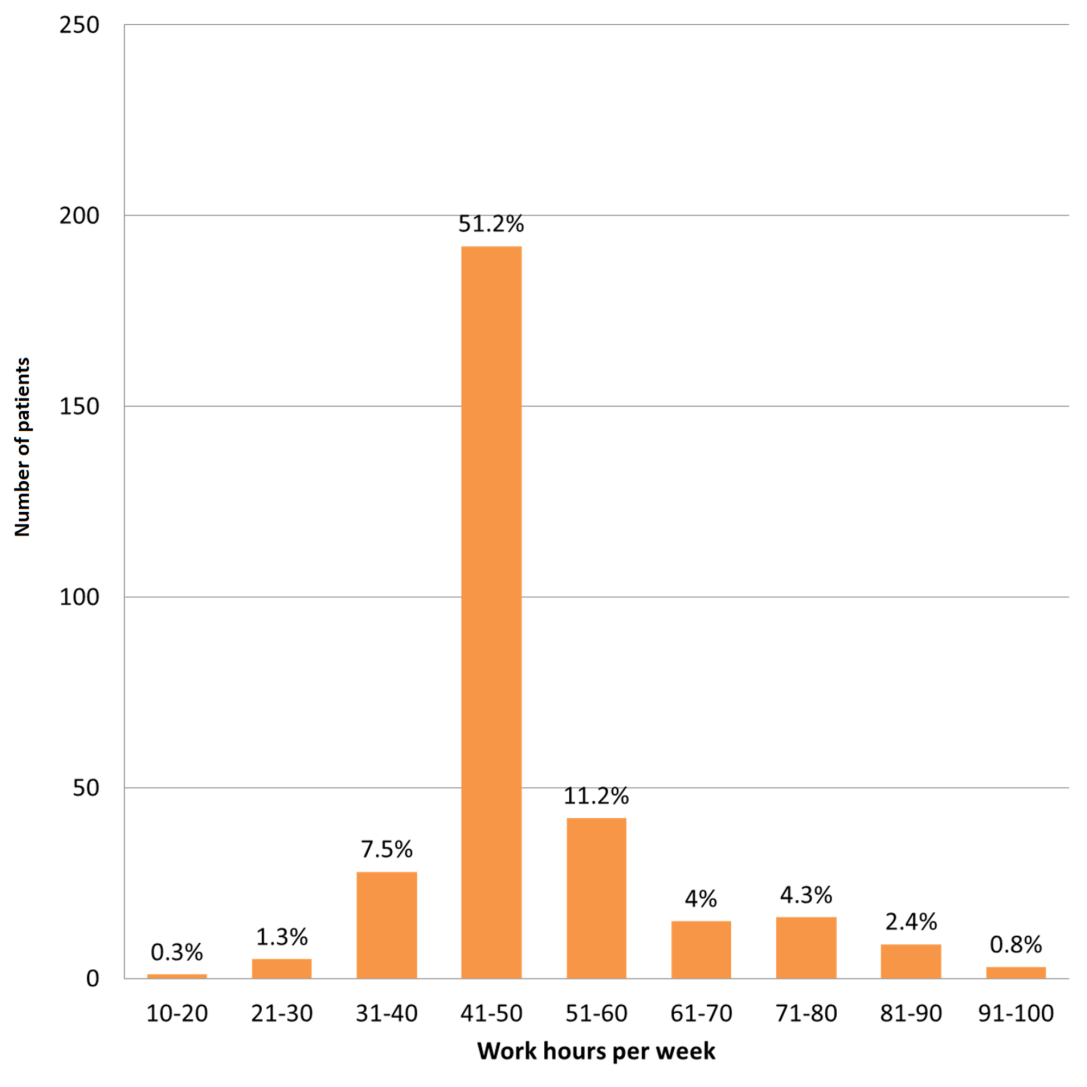
Distribution of work hours per week among patients with lower back pain.

Regarding work hours per week, 51.2% of patients reported working around 41–50 hours per week. Figure 3 illustrates the distribution of work hours per week among the patients with LBP.

**Figure 4:**
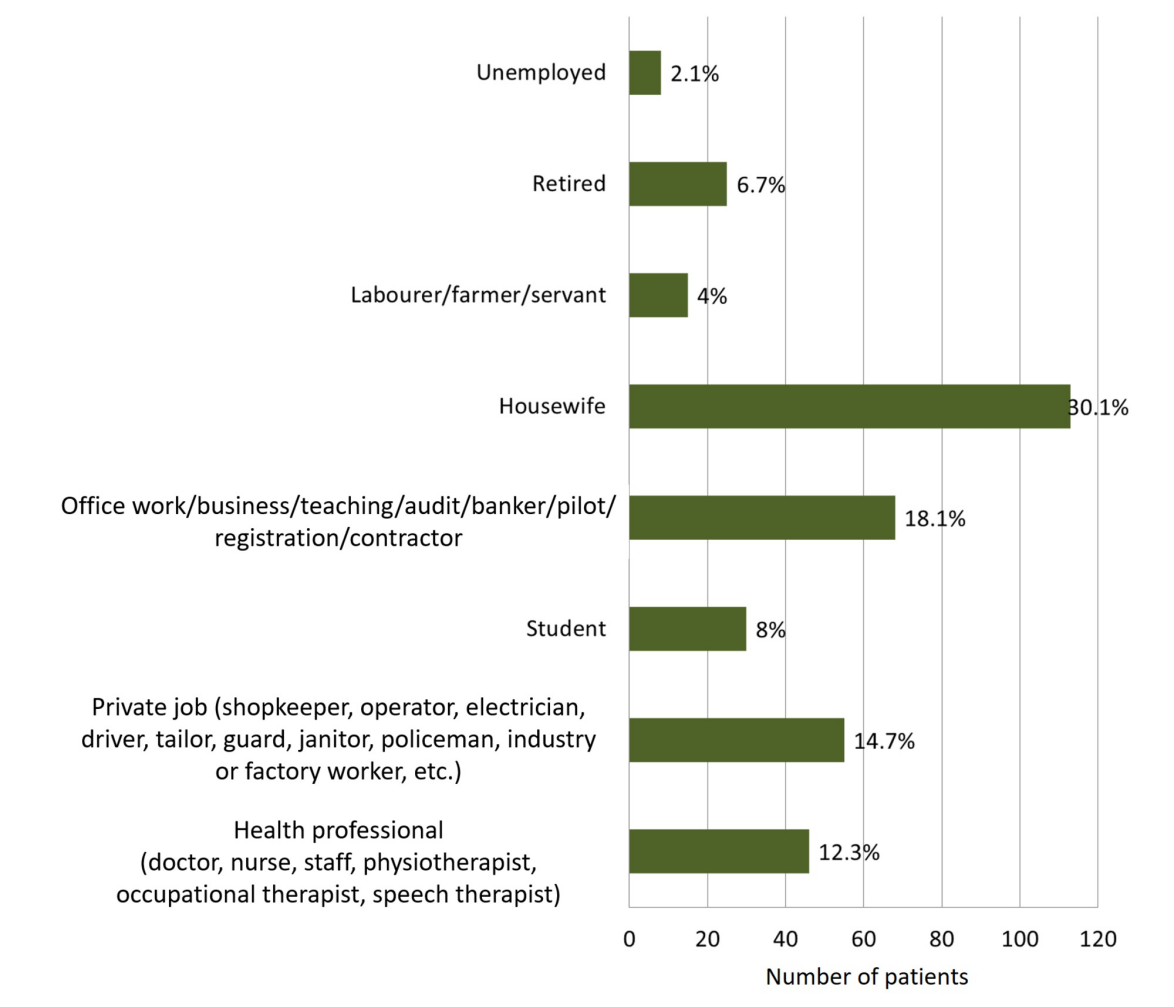
Distribution of lower back pain among different occupations.

Lower back pain was found to be predominant in housewives (30.1%, n = 113), followed by those with office jobs (18.1%, n = 68), private jobs (i.e., truck drivers, shopkeepers) (14.7%, n = 55), and healthcare workers (12.3%, n = 46) as shown in Figure 4.

The major risk factors were lack of exercise (76.3%, n = 286), use of soft foam mattress (52%, n = 195), prolonged sitting (50.4%, n = 189), lifting heavy weight (48.5%, n = 182), bending or twisting movement (41.6%, n = 156), sleep disorder (41.6%, n = 156), anxiety (39.5%, n = 148), hypertension (32.3%, n = 121), and depression (28.8%, n = 108). The frequency of risk factors correlating with back pain is shown in Table 1.

**Table 1:** Frequency of risk factors correlated with back pain.

	Back Pain Frequency, n (%)	
	Risk factor present	Risk factor absent
Smoking	62 (16.5%)	313 (83.5%)
Hypertension	121 (32.2%)	254 (67.7%)
Alcohol	6 (1.6%)	369 (98.4%)
Coronary artery disease	33 (8.8%)	342 (91.2%)
Dyslipidemia	68 (18.1%)	307 (81.9%)
Trauma	98 (26.1%)	277 (73.8%)
Prolonged sitting	189 (50.4%)	186 (49.6%)
Weightlifting	182 (48.5%)	193 (51.5%)
Bending	156 (41.6%)	219 (58.4%)
Sleep disorder	156 (41.6%)	219 (58.4%)
Sleeping in prone position	66 (17.6%)	309 (82.4%)
Soft foam mattress	195 (52.0%)	180 (48.0%)
Exercise	89 (23.7%)	286 (76.3%)
Anxiety	148 (38.5%)	227 (60.5%)
Depression	108 (28.8%)	267 (71.2%)
Family imbalance	82 (21.9%)	293 (78.1%)
Work environment	72 (19.2%)	303 (80.8%)
Job insecurity	48 (12.8%)	327 (87.2%)

## Discussion

LBP is one of the most common causes of doctor visits and has a global prevalence estimated at 9.4% [5]. We decided to look into various risk factors and their frequency in patients with lower back pain. The goal was to be able to see what risk factors are more frequently seen in Pakistani patients so that it can simplify both the early identification and management of those risk factors in our local population.

We noticed that 121 of 375 patients (32.3%) in our sample with LBP also had associated hypertension, defined as systolic blood pressure ≥ 140 mmHg and diastolic blood pressure ≥ 90 mmHg. In the literature, hypertension has been associated with a low prevalence of back pain; this is possibly due to hypalgesia [11].

LBP with comorbid coronary artery disease (CAD) was present in 33 of 375 patients (8.8%) while LBP with dyslipidemia was present in 68 of 375 patients (18.1%). While it is believed that there is no etiological link between LBP and CAD [12], dyslipidemia may lead to LBP due to aortic atherosclerosis [13].

LBP with a history of lower back trauma was seen in 98 of 375 patients (26.1%). There is a lack of literature establishing a correlation between lower back trauma and LBP in Pakistan.

Among the patients, 189 of 375 (50.4%) reported LBP with a history of prolonged unsupported sitting. This is believed to be due to changes in the L4–L5 vertebra that may contribute to symptoms [14]. In Pakistan, workplace ergonomics are inadequately practised. According to our study, 114 of 375 patients (30.4%) with LBP worked at different office and desk jobs or were employed in the healthcare environment. Additionally, 113 of 375 patients (30.1%) with LBP were identified as housewives. In Pakistan, a housewife is expected to undergo tiring routines involving hand washing clothes and dishes, cooking and cleaning—all without any care given to posture or comfort.

We found that 286 of 375 patients (76.3%) with LBP did not exercise. Exercise is generally believed to decrease the prevalence of LBP [15]. We observed that 195 of 370 patients (52%) with LBP used a soft foam mattress while 180 of 375 (48%) used a hard foam mattress. There is no concrete evidence on the type of mattress preferred for avoiding or treating LBP and there are differences between the American, Canadian and European guidelines on the topic. However, it is generally believed that medium-firm mattresses are better than soft foam or very hard mattresses [16].

It was noted that 62 of 375 patients (16.5%) with LBP also smoked. We did not differentiate current from former smokers. It is believed that smokers have a higher incidence and prevalence of LBP compared to people who have never smoked [17]. We found that six of 375 patients (1.6%) with LBP consumed alcohol. This is consistent with the estimated prevalence of heavy episodic drinking of 0.1% and the prevalence of alcohol dependence in both genders of 0.3% in Pakistan [18].

Heavy weight lifting was reported by 182 of 375 patients (48.5%). However, the weight, duration and nature (manual labour versus recreational weightlifting) of the heavy weight lifting was not defined.

The most common risk factors in the psychosocial category were sleep disorder, seen in 156 of 375 patients (41.6%), anxiety in 148 of 375 patients (39.5%), depression in 108 of 375 patients (28.8%), family disputes in 82 of 375 patients (21.9%), stress at the workplace in 72 of 375 patients (19.2%), and job insecurity in 48 of 375 patients (12.8%).

On the basis of our results, we believe that patients complaining of LBP should have a detailed social history work up that asks about their work and how often they sit unsupported. Likewise, homemakers should be given particular attention as the lack of ergonomic support as they go about their daily routines may be contributing to LBP. Similarly, exercise should be offered as a suggestion as good practice, even to otherwise healthy patients, owing to its protective role in the development of LBP and other problems later on. Similarly, smoking status should also be inquired about, and patients counselled to quit owing to its range of disastrous effects including LBP. Heavy lifting related LBP can be avoided by physicians advising patients on correct lifting techniques to prevent LBP.

When it comes to psychosocial problems, it can be seen that sleep disorders require careful analysis and management by the physician. Anxiety and depression should also be kept in mind as they may manifest as LBP.

## Conclusions

Our study concludes that lower back pain is a multifactorial phenomenon. Age, gender, profession, working hours, comorbid conditions, trauma, lifestyle, stresses in life all play a role in its causation. It is suggested to increase physical activity and modify lifestyle to prevent this major health issue.
